# Gallic acid improves the metformin effects on diabetic kidney disease in mice

**DOI:** 10.1080/0886022X.2023.2183726

**Published:** 2023-03-02

**Authors:** Yan Hong, Jidong Wang, Wenjuan Sun, Lai Zhang, Xuefang Xu, Kaiyue Zhang

**Affiliations:** a Department of Nephrology, Jiangnan University Medical Center (JUMC), Wuxi, China; b Department of Nephrology, Wuxi No. 2 People’s Hospital, Affiliated Wuxi Clinical College of Nantong University, Wuxi, China

**Keywords:** Gallic acid, metformin, diabetes mellitus, nephropathy

## Abstract

**Objectives:**

Metformin is an antidiabetic agent that is used as the first-line treatment of type 2 diabetes mellitus. Gallic acid is a type of phenolic acid that has been shown to be a potential drug candidate to treat diabetic kidney disease, an important complication of diabetes. We aimed to test whether a combination of gallic acid and metformin can exert synergetic effect on diabetic kidney disease in diabetic mice model.

**Methods:**

Streptozotocin (65 mg/kg) intraperitoneal injection was used to induce diabetic kidney disease in mice. The diabetic mice were treated with saline (Vehicle), gallic acid (GA) (30 mg/kg), metformin (MET) (200 mg/kg), or the combination of gallic acid (30 mg/kg) and metformin (200 mg/kg) (GA + MET).

**Results:**

Our results demonstrated that compared to the untreated diabetic mice, all three strategies (GA, MET, and GA + MET) exhibited various effects on improving renal morphology and functions, reducing oxidative stress in kidney tissues, and restoring AMP-activated protein kinase (AMPK)/silent mating type information regulation 2 homolog 1 (SIRT1) signaling in kidney tissues of diabetic mice. Notably, the combination strategy (GA + MET) provided the most potent renal protection effects than any single strategies (GA or MET).

**Conclusion:**

Our results support the hypothesis that gallic acid might serve as a potential supplement to metformin to enhance the therapeutical effect of metformin.

## Introduction

Diabetes is associated with various vascular complications and is recognized as one of the major causes of death worldwide, according to statistical data from the World Health Organization (WHO) [1–3]. Diabetic kidney disease is commonly observed in diabetes mellitus patients. Over the years, unwell controlled diabetes cause damage to the blood vessel in kidneys leading to impaired renal morphology and functions. The pathophysiological characterization of diabetic kidney disease includes glomerular hyperfiltration, elevated serum urinary albumin, mesangial cell expansion, tubulointerstitial fibrosis, and renal hypertrophy [4,5].

Metformin is a Food and Drug Administration (FDA)-approved antidiabetic agent for the treatment of patients with type 2 diabetes mellitus [6]. Although metformin is one of the antidiabetic drugs that are usually effective in slowing down the ongoing deterioration of diabetes, its effect is less optimal to reverse the progression of diabetic kidney disease in the long run [7]. Therefore, the use of combination of metformin and natural bioactive compound to treat or maintain diabetic kidney disease has gained increasing attention [8]. A recent study reported that addition of lycopene, a type of carotenoid found in vegetables and red fruits, improves the glycemic control effect of metformin on diabetic rats by decreasing glycoxidative stress [9].

It is reported that metformin is able to inhibit hepatic glucose production through the suppression of gluconeogenesis [10]. Further studies revealed that metformin enters hepatocytes and partially blocks the mitochondrial respiratory chain complex I, resulting in inhibiting adenosine triphosphate (ATP) production. The decreased ATP levels and increased adenosine monophosphate (AMP) activates the AMP-activated protein kinase (AMPK) signaling pathway, which in turn suppresses the transcription of gluconeogenic genes [11,12]. Enhancing AMPK signaling boosts glucose in diabetes, which is important for reducing hyperglycemia [13]. Further analysis indicates the function of AMPK signaling in improving insulin sensitivity for diabetic patients’ treatment [14] Furthermore, clinical research on diabetes patients have demonstrated the involvement of AMPK signaling in the amelioration of diabetic sequelae such as nephropathy, brain disorder, and liver disease [15]. As a result, therapies based on AMPK targeting may be effective in the treatment of diabetes. How the AMPK signaling pathway is affected under the influence of various agents (e.g., metformin and gallic acid) in a diabetic mouse model is not fully understood.

Although metformin is normally effective, diabetes is a progressive condition with continual degradation of energy metabolism regulation, and metformin therapy may fail to provide long-term glycemic control and to reverse the course of diabetic complications [7]. In this light, it has attracted attention that combining therapy involving natural bioactive compounds could dramatically improve glycemic control in diabetes mellitus while also helping to minimize the complications associated with the condition. Gallic acid is a type of phenolic acid that commonly presents in many food materials and medicinal plants. Gallic acid treatment has been shown to mitigate type 1 diabetic kidney disease in streptozotocin-induced diabetic rats [16,17]. Inspired by these findings, we hypothesized that combination of gallic acid and metformin might exert synergistic effect on renal protection in diabetic kidney disease. This study was carried out to address our hypothesis.

## Materials and methods

### Diabetic mouse model

The diabetic mouse model was established following the procedures as described previously [18]. Eight-week-old C57BL/6J male mice were purchased from GemPharmatech (Nanjing, China). The animals were housed in a clear animal facility with a 12 h light and dark cycle. Mice were acclimatized to the environment of animal facility for at least one week before experiment with free access to food and water. The animal experiment protocol was approved by the Ethics Committee of Wuxi No.2 People’s Hospital (#2021.03.e8). Mice were fasted overnight and were injected with a single intraperitoneal (i.p.) injection of streptozotocin (65 mg/kg) in the early morning [18]. Streptozotocin injection solution was diluted in sodium citrate buffer (0.1 M, pH 4.5). Mouse blood samples were collected three days poststreptozotocin injection. The blood glucose levels were assessed using a portable glucometer (ADVANTAGE, Boehringer Mannheim, MO, USA). Only those mice with high fasting glucose levels were selected for the following study.

The diabetic mice were randomly divided into four groups with 26 mice in each group. In each group, 18 mice were for three different kinds of tissue staining with six mice for each staining (HE staining, Periodic Acid Schiff (PAS) staining and dihydroethidium (DHE) staining), and another eight mice were for tissue homogenate for oxidative stress assay and Western blot experiments. The untreated normal mice were assigned as normal group. Diabetic mice were respectively treated with gallic acid (GA) (purity ≥ 98%, Sigma, St Louis, MO, USA) at 30 mg/kg, [16,18] metformin (MET) at 200 mg/kg, [19] or a combination of GA (30 mg/kg) and MET (200 mg/kg) daily for a total of four weeks. The diabetic mice treated with saline were assigned as Vehicle group. The mouse body weight and fasting blood glucose were measured every week. After four weeks, the animals were anesthetized with halothane then submitted to euthanasia. Kidney to body weight values were calculated by the weight of kidney divided by the weight of mouse body. For biochemical tests, samples of kidney were placed on ice and homogenized in 50 mM Tris-HCl pH 7.4 (1/10, w/v) and stored at −80 °C until usage. For histopathological analysis, the samples of kidney were fixed with 4% paraformaldehyde.

### Determination of serum parameters

Mouse blood samples were collected at the end of experiments. Serum urea, urea nitrogen (BUN), and creatinine were respectively assessed by Urea assay kit (MAK006, Sigma-Aldrich, St. Louis, MO, USA), Urea Nitrogen (BUN) colorimetric detection kit (EIABUN, Thermo Fisher Scientific, Waltham, MA), and Creatinine assay kit (MAK080, Sigma-Aldrich) according to the manufacturer’s instructions.

### Determination of urine parameters

Mouse blood samples were collected using metabolic cages at the end of experiment. Total urine volume was collected for a total of 24 h. Urine albumin and urine total protein were respectively assessed by BCG albumin assay kit (MAK124, Sigma-Aldrich) and urine total protein kit (TP0100, Sigma-Aldrich) according to the manufacturer’s instructions. The creatinine clearance was calculated using the following formula: creatinine clearance = Urine creatinine × Urine volume (mL)/Plasma Creatinine × Time (Min).

### Periodic acid Schiff (PAS) staining and hematoxylin and eosin (H&E) staining

Kidney tissues were fixed in formalin, and the paraffin blocks were sliced into 4 µm sections. The kidney section was stained with PAS stain Kit (395B, Sigma-Aldrich) or H&E staining Kit (ab245880, Abcam, Cambridge, MA). Glomeruli area and mesangial matrix area were observed under a digital microscope. The mesangial matrix index was calculated by 100 × mesangial matrix area/glomeruli area. For assessing H&E staining results, the following factors were used to estimate tubulointerstitial lesions for HE staining: a, atrophy, expansion, and casts of tubules; b, tubulointerstitial fibrosis and infiltration of inflammatory cells. The specific grades varied from zero to four points: zero for no changes, one for changes of 25% or less in the section, two for changes of 25–50%, three for changes of 50–75%, and four for changes of 75–100%.

### Western blotting

Western blotting assay was performed by the method described by Li et al. [20]. Briefly, the proteins were separated by sodium dodecyl sulfate polyacrylamide gel electrophoresis (SDS-PAGE) and were transferred onto a PVDF (polyvinylidene difluoride) membrane. Immunoblot assay was performed by using anti-p-AMPK (#ab133448, rabbit monoclonal [CAT No. EPR5683] to AMPK alpha 1 (phospho T183) + AMPK alpha 2 (phospho T172), 1:1000), anti-AMPK (#ab207442, rabbit monoclonal [CAT No. EPR19549] to AMPK alpha 1 + AMPK alpha 2, 1:1000), anti-SIRT1 (#ab110304, 1.25 µg/mL), and anti-GAPDH (#ab8245, 1:1000) from Abcam. Protein signals were detected using enhanced chemiluminescence (ECL).

### The DHE (dihydroethidium) staining

The frozen kidneys were placed on a slide and embedded in OCT compound at −20 °C. The frozen renal tissues were further cut into 5 μm thick sections using a cryostat microtome (Leica microsystem, Wetzlar, Germany). After washing to get rid of OCT, the tissues on the slide were stained with 5 μM DHE solution for 15 min in a dark room. After washing the dyed slides, a fluorescence microscope was used to capture the pictures (Evos imaging fluorescence microscope, Thermo Fisher Scientific).

### Statistical analysis

Data are presented as mean ± standard deviation (SD). Anderson–Darling test, D’Agostino and Pearson test, Shapiro–Wilk test and Kolmogorov–Smirnov test were used to test the normality of the data. The statistical differences between the experimental groups were assessed by ANOVA analysis with *post hoc* tests of Dunnett’s T3 multiple comparisons test or Turkey’s multiple comparisons test. *p* values less than 0.05 were considered to be significant.

## Results

### Combination of GA and MET improves body weight and reduces fasting blood glucose levels in diabetic mice

**Figure 1.**
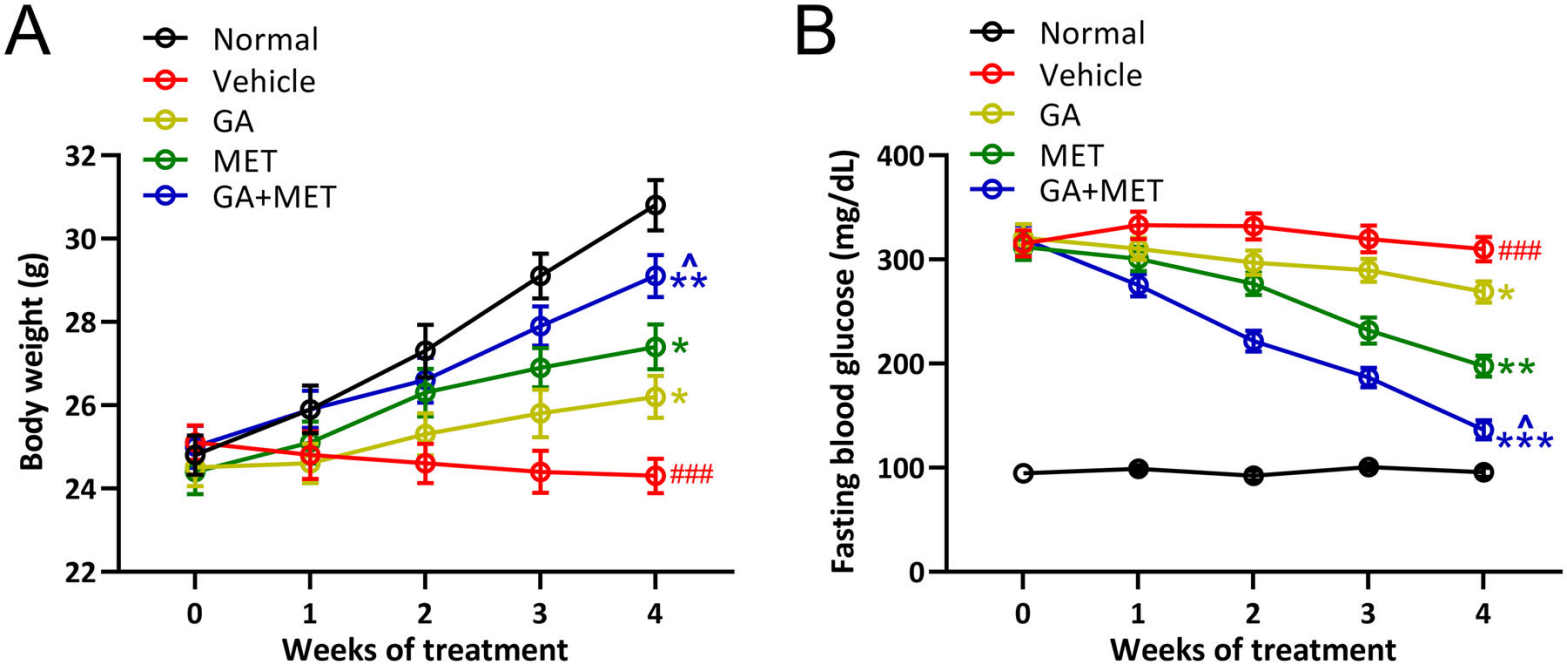
Effects of metformin combined with gallic acid treatment on body weight (A) and fasting blood glucose levels (B) in mice model of diabetic nephropathy. All mice had free access to food and water at all times. Fasting blood glucose levels and body weight were measured once a week. 10 mice were used for each group. Data are presented as mean ± *SD*. ^###^*p* < 0.001 compared to normal. *p < 0.05, **p < 0.01 compared to vehicle. ^^^*p* < 0.05 compared to MET group.

Diabetic mice exhibited significant body weight loss and elevated fasting blood glucose levels compared with normal mice. As shown in Figure 1(A,B), the body weight of normal mice gradually increased, whereas the body weight of diabetic mice slowly decreased in a time-dependent manner. Similarly, the fasting blood glucose levels were consistently over three-fold higher in diabetic mice compared to control mice. Interestingly, we observed that gallic acid-alone (GA), metformin-alone (MET), or a combination of gallic and metformin (GA + MET) treatment gradually increased body weight (Normal: 30.82 ± 1.91 g, Vehicle: 24.33 ± 1.31 g, GA: 26.18 ± 1.63 g, MET: 27.38 ± 1.74 g, GA + MET: 29.13 ± 1.64 g. After 4 weeks of treatment) and reduced fasting blood glucose levels (Normal: 95.64 ± 13.56 mg/dL, Vehicle: 309.72 ± 36.77 mg/dL, GA: 268.68 ± 31.92 mg/dL, MET: 197.51 ± 31.48 mg/dL, GA + MET: 136.33 ± 28.56 mg/dL. After 4 weeks of treatment) in diabetic mice suggesting GA, MET, or GA + MET treatment is able to alleviate the adverse effects of diabetes in mice. Although MET showed stronger improvement effect than GA, the GA + MET revealed the most potent effect among three treatment groups in improving body weight and reducing fasting blood glucose levels in diabetic mice.

### Combination of GA and MET improves renal functions

**Figure 2.**
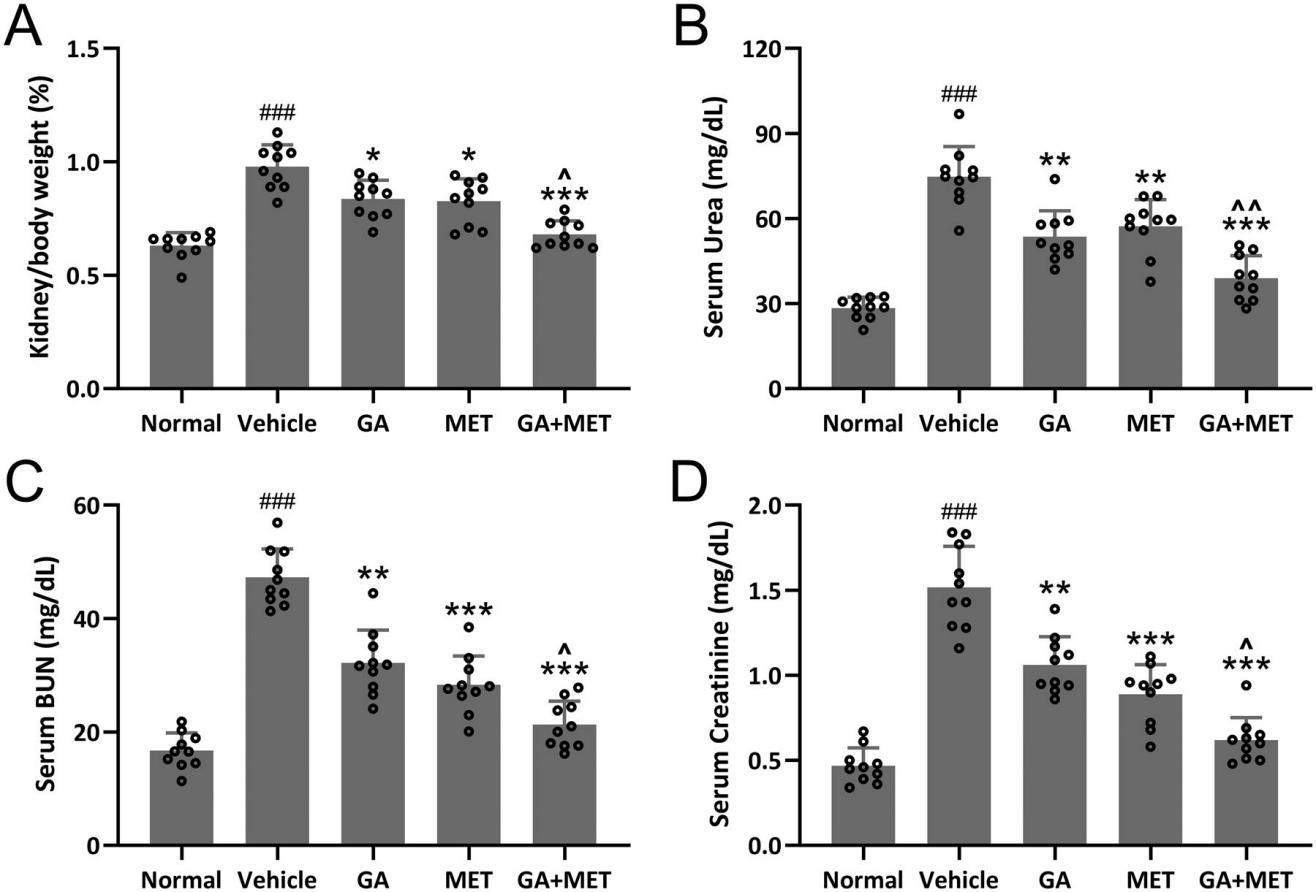
Effects of metformin combined with gallic acid treatment on kidney/body weight (A), Urea (B), BUN (C) and Creatinine (D) in serum of mice model of diabetic kidney disease at the end of 4 weeks treatment. Serum levels of blood urea nitrogen (BUN), urea, and creatinine (Cr) were analyzed by an automatic analyzer (cobas^®^ 8000 modular analyzer series. Roche Diagnostics). 10 mice were used for each group. Data are presented as mean ± SD. ^###^*p* < 0.001 compared to normal. *p < 0.05, **p < 0.01, ***p < 0.001 compared to vehicle. ^*p* < 0.05, ^^*p* < 0.01 compared to MET group.

To explore effect of these drugs on improving kidney function in diabetic mice, the kidney to body ratio, serum urea, serum BUN, and serum creatinine were measured in five groups of mice. As expected, the value of kidney to body ratio and the levels of urea, BUN, and creatinine in serum were significantly higher in diabetic mice compared with normal mice (Figure 2(A–D)). GA, MET, or GA + MET treatment was capable of reducing the value of kidney to body ratio and the levels of urea, BUN, and creatinine in serum. The effect of MET and GA seems comparable, while MET + GA exhibited the most substantial effect on the restoration of kidney to body ratio, serum urea (Normal: 28.45 ± 3.82 mg/dL, Vehicle: 74.76 ± 10.61 mg/dL, GA: 53.62 ± 9.12 mg/dL, MET: 57.24 ± 9.43 mg/dL, GA + MET: 38.92 ± 7.91 mg/dL.), serum BUN (Normal: 16.73 ± 3.11 mg/dL, Vehicle: 47.28 ± 5.01 mg/dL, GA: 32.19 ± 5.78 mg/dL, MET: 28.31 ± 5.11 mg/dL, GA + MET: 21.31 ± 4.14 mg/dL.), and serum creatinine (Normal: 0.47 ± 0.11 mg/dL, Vehicle: 1.52 ± 0.24 mg/dL, GA: 1.06 ± 0.17 mg/dL, MET: 0.89 ± 0.17 mg/dL, GA + MET: 0.62 ± 0.13 mg/dL.) in diabetic mice (Figure 2(A–D)). In addition, MET combined with GA treatment could increase the serum insulin and HDL levels, and decrease the serum TC and TG levels when compared to Vehicle group (Figure S1).

### Combination of GA and MET exerts renal protective effect

**Figure 3.**
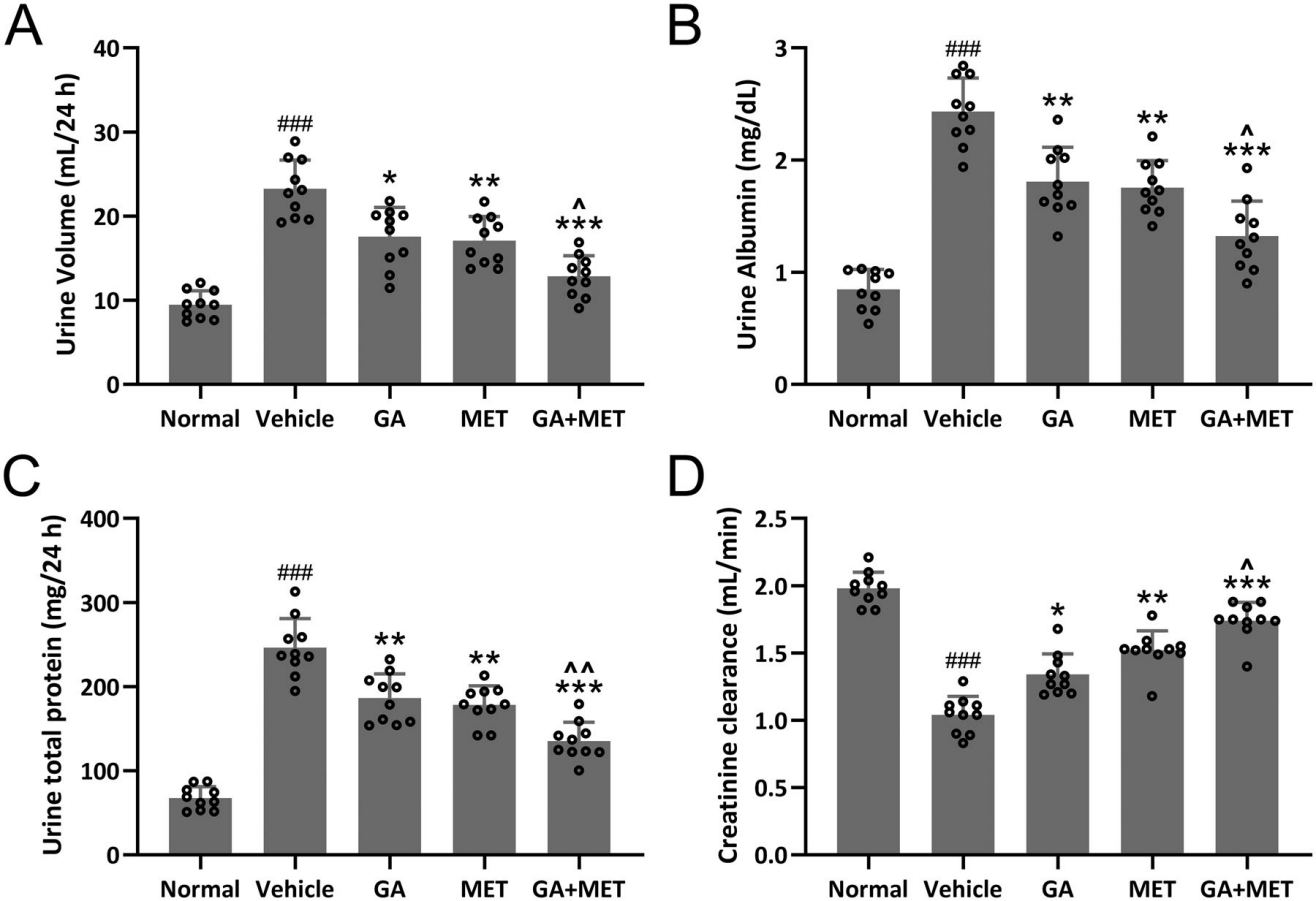
Effects of metformin combined with gallic acid treatment on urine volume (A), urine albumin (B), total proteins (C) and Creatinine clearance (D) in urine of mice model of diabetic kidney disease at the end of 4 weeks treatment. Urinary levels of albumin, total proteins, and creatinine were analyzed by an automatic analyzer (cobas^®^ 8000 modular analyzer series. Roche Diagnostics). 10 mice were used for each group. Data are presented as mean ± *SD*. ^###^*p* < 0.001 compared to normal. *p < 0.05, **p < 0.01, ***p < 0.001 compared to vehicle. ^*p* < 0.05, ^^*p* < 0.01 compared to MET group.

To explore effect of these drugs on kidney protection in diabetic mice, the urine volume, urine albumin, urine total protein, and creatinine clearance were assessed in five groups of mice. As depicted in Figure 3(A–D), the values of urine volume, urine albumin, urine total protein were significantly enhanced, whereas the values of creatinine clearance were markedly decreased in diabetic mice compared with normal mice, implying impaired kidney function in diabetic mice compared to normal mice. The results in Figure 3(A–D) showed that all three treatments (GA, MET, or GA + MET) exerted kidney protective effect on diabetic mice as demonstrated by reduced the values of urine volume (Normal: 9.46 ± 1.65 mL/24 h, Vehicle: 23.26 ± 3.42 mL/24 h, GA: 17.55 ± 3.51 mL/24 h, MET: 17.08 ± 2.89 mL/24 h, GA + MET: 12.85 ± 2.45 mL/24 h.), urine albumin (Normal: 0.85 ± 0.18 mg/dL, Vehicle: 2.43 ± 0.29 mg/dL, GA: 1.81 ± 0.31 mg/dL, MET: 1.76 ± 0.24 mg/dL, GA + MET: 1.32 ± 0.31 mg/dL.), urine total protein (Normal: 67.46 ± 13.65 mg/24 h, Vehicle: 246.41 ± 34.45 mg/24 h, GA: 186.52 ± 28.92 mg/24 h, MET: 178.24 ± 22.71 mg/24 h, GA + MET: 135.48 ± 22.17 mg/24 h.), and increased values of creatinine clearance (Normal: 1.98 ± 0.12 mL/min, Vehicle: 1.04 ± 0.14 mL/min, GA: 1.34 ± 0.15 mL/min, MET: 1.52 ± 0.15 mL/min, GA + MET: 1.74 ± 0.13 mL/min.). The GA + MET exhibited the most potent renal protective effect among three treatments.

### Combination of GA and MET reduces renal damage

**Figure 4.**
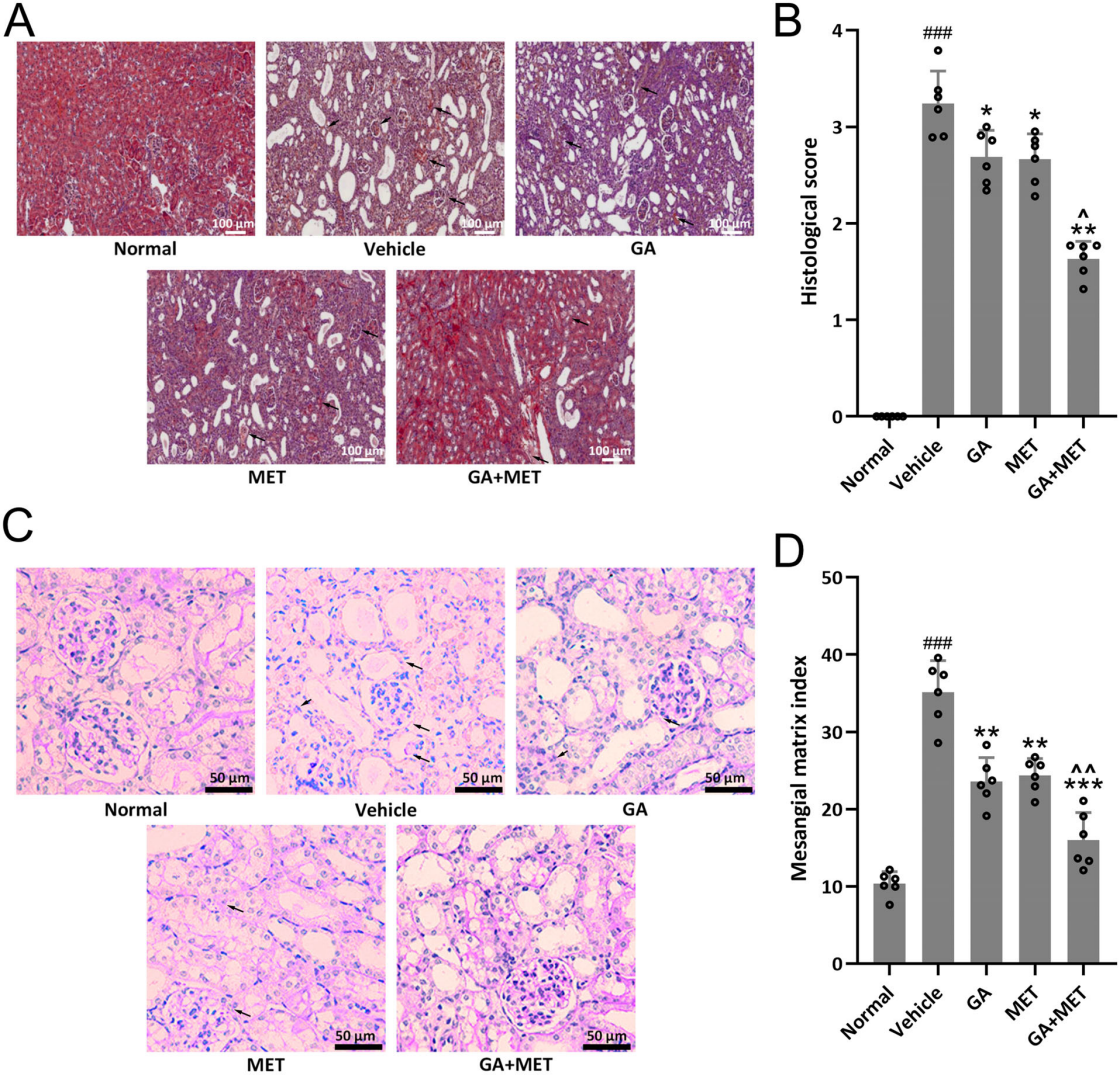
Effects of metformin combined with gallic acid treatment on histopathological change of the kidney at the end of 4 weeks treatment. (A) Representative HE staining among different groups and the quantification of histological score (B). The data were gotten from six mice in each group and the data point indicates the average of single mouse from eight random fields. (C) Representative Periodic Acid Schiff (PAS) staining among different groups and the quantification of mesangial matrix index (D). The data were gotten from six mice in each group and the data point indicates the average of single mouse from 10 random fields. Data are presented as mean ± SD. ^###^*p* < 0.001 compared to normal. *p < 0.05, **p < 0.01, ***p < 0.001 compared to vehicle. ^*p* < 0.05, ^^*p* < 0.01 compared to MET group.

The kidney protective effect results were further confirmed by PAS staining assay. As shown the Figure 4(A,B), the tubulointerstitial lesions as determined by H&E staining and the tubulointerstitial lesion status were assessed by histological score. The histological score was 3.2 in renal tissues from diabetic mice, while 0 for renal tissues from normal mice. The histological score for renal tissues from GA-, MET-, or GA + MET-treated diabetic mice was 2.8, 2.7, and 1.6, respectively. Furthermore, mesangial matrix index assessed by PAS staining was significantly enhanced in diabetic mice than normal mice and was markedly reduced in the GA-, MET-, or GA + MET-treated diabetic mice (Figure 4(C,D)). These results suggest that the diabetic mice showed more severe glomerular damage than that in normal mice, and GA-, MET-, or GA + MET-treatment displayed kidney protective effect, in which GA + MET showed the strongest effect, on diabetic mice.

### Combination of GA and MET decreases oxidative stresses

**Figure 5.**
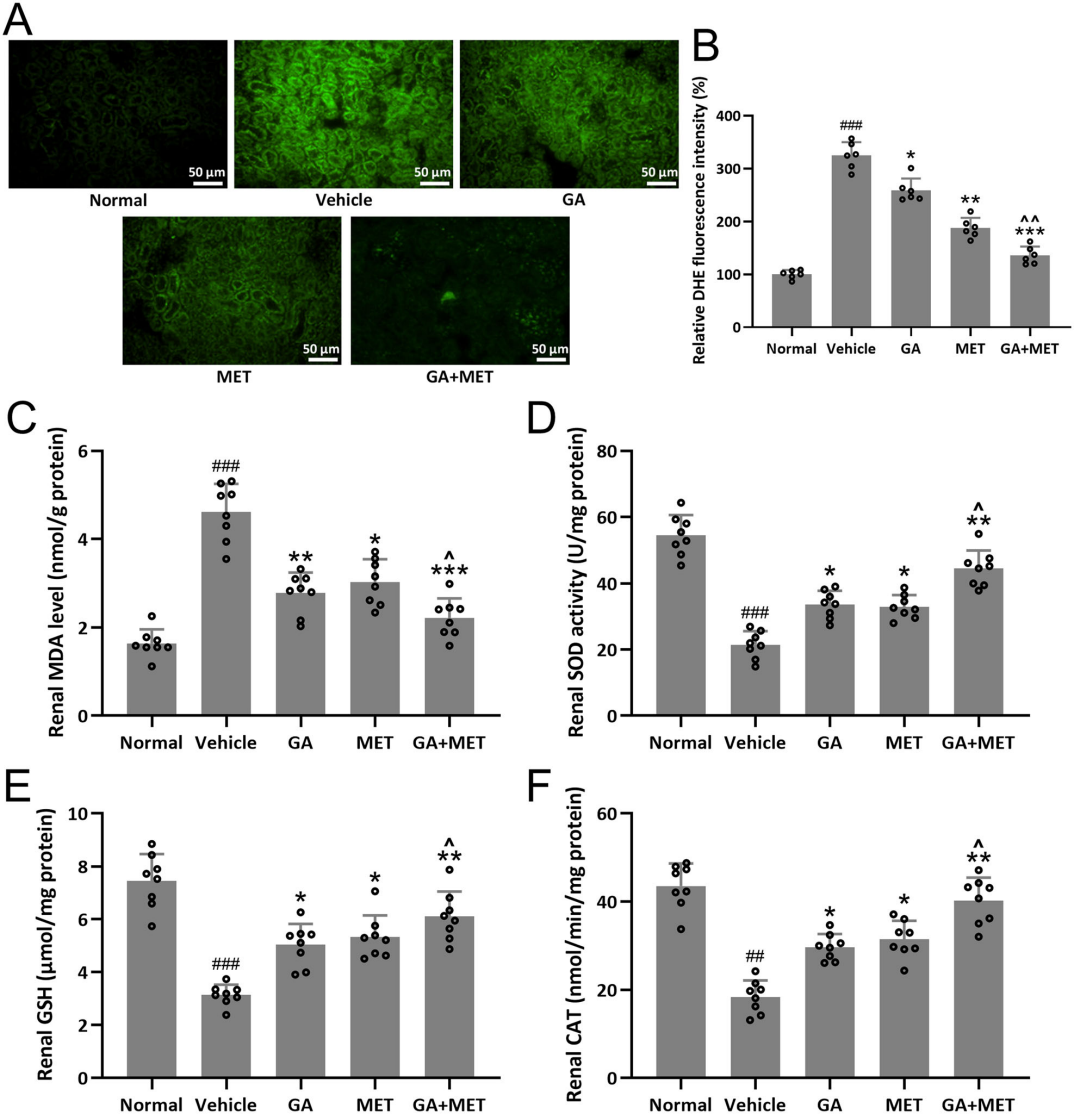
Effects of metformin combined with gallic acid treatment on renal oxidative stresses in mice model of diabetic kidney disease at the end of 4 weeks treatment. (A) Representative images of dihydroethidium (DHE) staining for the assessment of superoxide production and (B) bar diagram showing the percent of DHE fluorescence intensity. The data were gotten from six mice in each group and the data point indicates the average of single mouse from eight random fields. The levels of MDA (C), SOD (D), GSH (E) and CAT (F) in renal tissues were measured. Eight mice were used for each group. Data are presented as mean ± *SD*. ^##^*p* < 0.01, ^###^*p* < 0.001 compared to normal. *p < 0.05, **p < 0.01, ***p < 0.001 compared to vehicle. ^*p* < 0.05, ^^*p* < 0.01 compared to MET group.

Oxidative stress augmentation is commonly observed in diabetic renal tissues [21,22]. The renal oxidative stresses levels were assessed using DHE staining of frozen kidney tissues. The results in Figure 5(A,B) showed that the ROS levels in diabetic renal tissues were substantially higher than normal renal tissues. Not surprisingly, we observed upregulation of MDA expression levels and downregulation of GSH expression, CAT expression, and SOD activity levels in diabetic renal tissues compared to normal renal tissues. GA-, MET-, or GA + MET-treatment was able to restore oxidative stress by restoring MDA, GSH, and CAT levels and SOD activity in diabetic renal tissues, with the most effective effect observed in GA + MET treatment (Figure 5(C–F)). Moreover, it was found that 10 mM gallic acid increased cell viability, and attenuated ischemia reperfusion (IR) injury-induced oxidative stress in human renal proximal tubular epithelial cell line (HK-2) (Figure S2(A–D)).

### Combination of GA and MET induces AMPK/SIRT1 signaling activation

**Figure 6.**
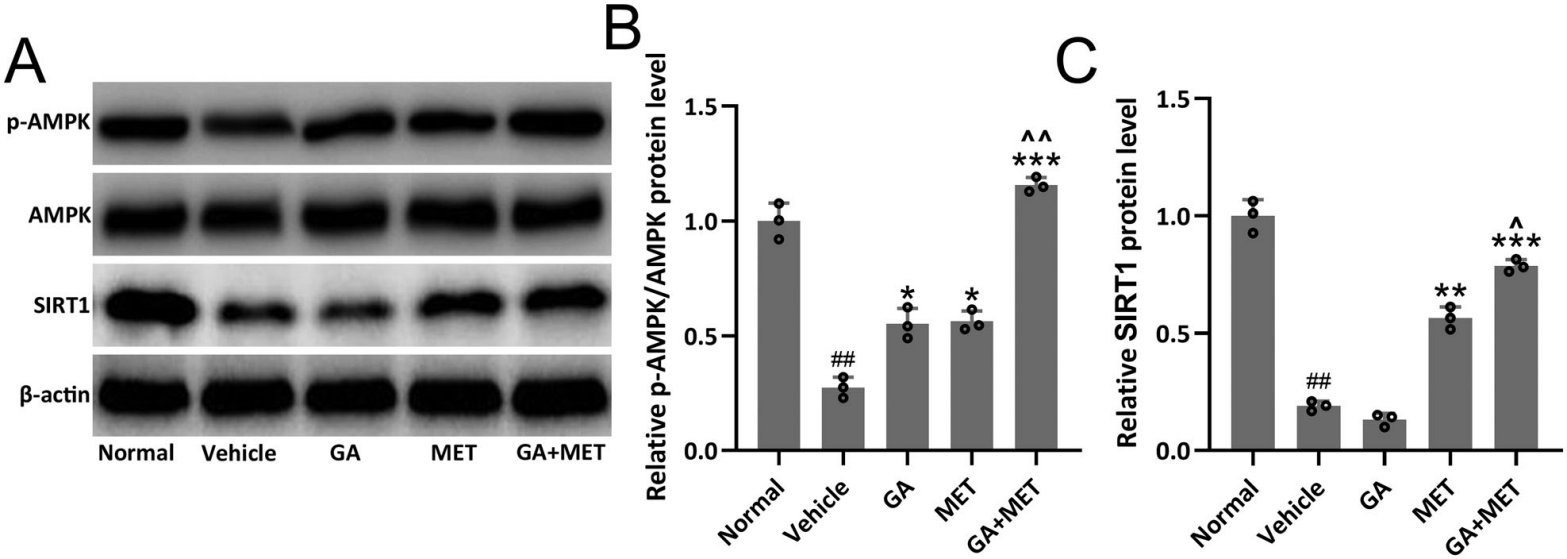
Effects of metformin combined with gallic acid treatment on renal AMPK/SIRT1 signaling pathway in mice model of diabetic nephropathy. Western blotting was used to measure the protein expressions of p-AMPK, AMPK and SIRT1 (A) and the relative expressions were normalized to normal (B and C). β-actin was used as a loading control. The data were gotten from three repeated experiments using renal homogenate from each group including eight mice. Data are presented as mean ± *SD*. ^##^*p* < 0.01, ^###^*p* < 0.001 compared to normal. *p < 0.05, **p < 0.01, ***p < 0.001 compared to vehicle. ^*p* < 0.05 compared to MET group.

AMPK/SIRT1 signaling pathway is known to play a critical role in diabetes development [23,24]. Significant downregulation of p-AMPK and SIRT1 levels was observed in diabetic renal tissues compared to normal renal tissues. GA or MET treatment only partially, whereas GA + MET treatment completely restored p-AMPK and SIRT1 levels in diabetic renal tissues, suggesting that GA + MET treatment potently induced AMPK/SIRT1 signaling activation (Figure 6(A–C)). Similarly, *in vitro* data from HK-2 cells demonstrated the activation of MPK/SIRT1 signaling pathway by 10 mM gallic acid treatment in the cells (supplemental Figure S3 (A–C)).

## Discussion

The current study was based on our hypothesis that combination of gallic acid and metformin may exert more potent renal protective effect than any single drug on diabetic mice. In this study, we applied the well-established streptozotocin injection to induce diabetic mice. Consistent with several previous publications, the diabetic control mice that received only vehicle displayed decreased body weight, and constant high fasting blood glucose levels when compared to normal mice, confirming the successful establishment of the streptozotocin-induced diabetic mice model [25]. In line with Mayuresh et al. [16] compared to the normal mice, the diabetic control mice exhibited significantly elevated kidney to body weight value, serum levels of Urea, BUN, and creatinine, urine volume, urine albumin, and total protein, and decreased creatinine clearance ability. These results suggested that renal function was severely damaged in diabetic mice. The PAS staining results exhibited that the mesangial matrix index, a method to evaluate glomerular destruction, [26,27] was considerably higher in diabetic mice than in normal mice. These results confirmed the occurrence of diabetic kidney disease in diabetic mice.

Meanwhile, we observed that GA, MET, or a combination of GA and MET (GA + MET) treatment was capable of attenuating or restoring diabetic kidney disease in diabetic mice. Because GA, MET, or GA + MET treated diabetic mice demonstrated various degrees of increased body weight, reduced high fasting blood glucose levels, enhanced renal function, and improved glomerular morphology. Although the renal protective effect was more robust in the MET-treated group than the GA-treated group, the combination groups (GA + MET) exhibited the most potent effect among the three groups, suggesting a synergistic protective effect was observed in the GA + MET group. In particularly, the increased glucose levels in diabetic condition would cause renal impairments [28]. Our results demonstrate that metformin combined with gallic acid could greatly reduce the levels of fasting blood glucose, which should contribute the beneficial role of GA and MET against the renal impairments. Interestingly, Mayuresh et al. and Shahnaz et al. reported that GA treatment alone exerted renal protective effect and improved renal function in diabetic rats and mice, respectively [16,29]. Our results not only confirmed their findings but also extended to a novel conclusion that additional GA might enhance the antidiabetic effect of MET, providing new insight into developing new methods for the treatment of diabetic patients.

It is known that increased serum/plasma glucose promotes overwhelming production of reactive oxygen species (ROS), which plays a critical role in diabetic complication pathogenesis [30,31]. NADPH oxidases (NOXs) and endothelial nitric oxide synthase (eNOS) can be activated by metabolic abnormalities of diabetic kidney disease, particularly hyperglycemia and hyperlipidemia, to impact oxidative stress [32]. Excessive oxidative production in the kidney may cause apoptosis in mesangial cells and tubular epithelial cells, resulting in the progression of diabetic nephropathy. Previous studies revealed that GA-alone or MET-alone treatment reduced ROS production and oxidative stress in kidney tissues [16,33]. Not surprisingly, we also observed the same phenotype in GA-alone or MET-alone treatment groups. The GA + MET group exhibited a more potent oxidative stress-reducing effect than the GA-alone or MET-alone group.

It has been reported that AMPK/SIRT1 signaling plays an important role in the development of diabetes. Recent study have demonstrated that enhancing SIRT1 antagonizes oxidative stress in the pathogenesis of diabetic kidney disease and that AMPK is an critical mediator that regulates SIRT1-mediated oxidative stress reduction [23,34–37]. We observed that GA or MET treatment only partially, whereas GA + MET treatment completely restored p-AMPK and SIRT1 levels in diabetic renal tissues, suggesting that GA + MET-mediated renal protective effect on diabetic mice through regulating AMPK/SIRT1 signaling activation.

As discussed above, the reduced fasting blood glucose and oxidative stress, and the activation of AMPK/SIRT1 signaling should contribute the protective effects of the combination of GA and MET treatment against renal impairments in diabetic mice. However, the causal relationship between glucose levels, oxidative stress and AMPK/SIRT1 signaling under the presence of GA and MET warrants further studies in the future.

### Limitation

This study was primarily focused on studying the potent renal protective effect of GA-alone or Met al.one, and the combination of GA and MET, on diabetic mice. However, their effect on the pancreas was not investigated, and the detailed molecular mechanisms for the reported protective effects were not delicately explored in this study. In addition, a dose escalating study on the combination of GA and MET is also highly desirable to find the optimal dose.

## Conclusion

In this study, we have compared the potential renal protection effects of three strategies (GA, MET, and GA + MET) on diabetic mice. Although previous studies have revealed that GA-alone or MET-alone treatment is beneficial for the management or treatment of diabetic animals and patients, our present study demonstrated that the combination of GA and MET exhibited a more potent renal protective effect in diabetic mice than GA-alone or MET-alone on enhancing renal morphology and functions, decreasing oxidative stress in kidney tissues, and restoring AMPK/SIRT1 signaling in kidney tissues of diabetic mice. These results suggest that supplemental GA might promote an antidiabetic effect of MET.

## Supplementary Material

Supplemental Material
