# Supplementary material for: Gallic acid improves the metformin effects on diabetic kidney disease in mice
Source: Ren Fail. 2023 Mar 2;45(1):2183726. doi: 10.1080/0886022X.2023.2183726 (PMC9987773; doi:10.1080/0886022X.2023.2183726)
Supplement: Supplemental Material [file IRNF_A_2183726_SM8951.pdf]

Supplementary materials

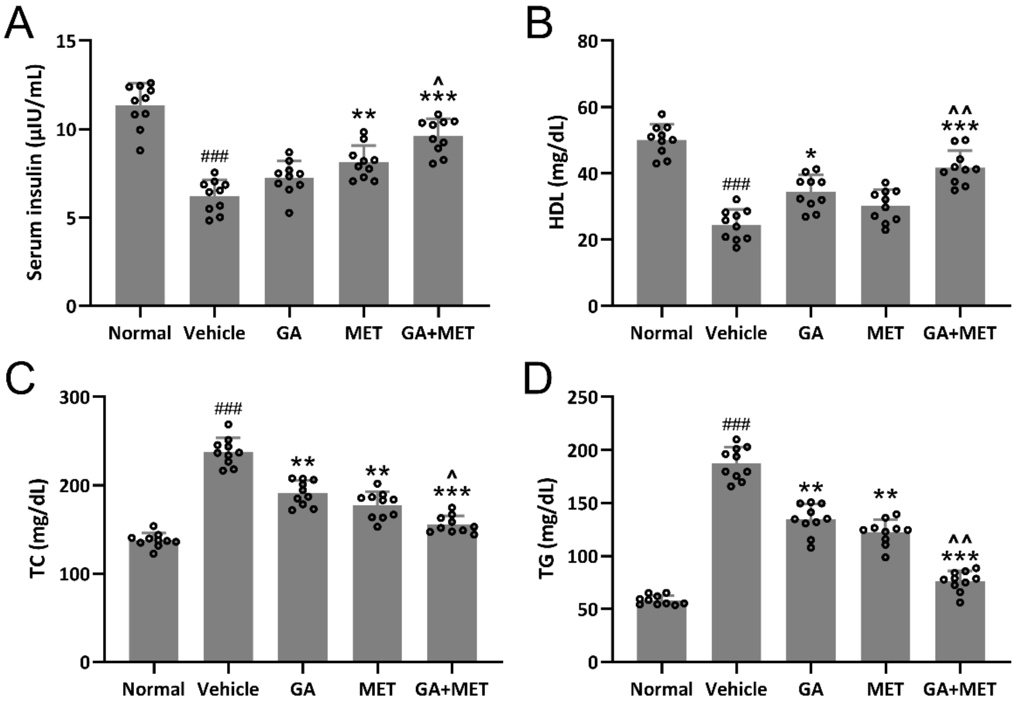

Figure S1. Effects of metformin combined with gallic acid treatment on glucose and lipid metabolism in mice model of diabetic kidney disease. The levels of serum insulin (A), HDL (B), TC (C) and TG (D)at the end of 4 weeks treatment. 10 mice were used for each group. Data are presented as mean ± SD. ###p < 0.001 compared to normal. *p < 0.05, **p < 0.01, ***p < 0.001 compared to vehicle. ^p < 0.05, ^^p < 0.01 compared to MET group.

Information Classification: General

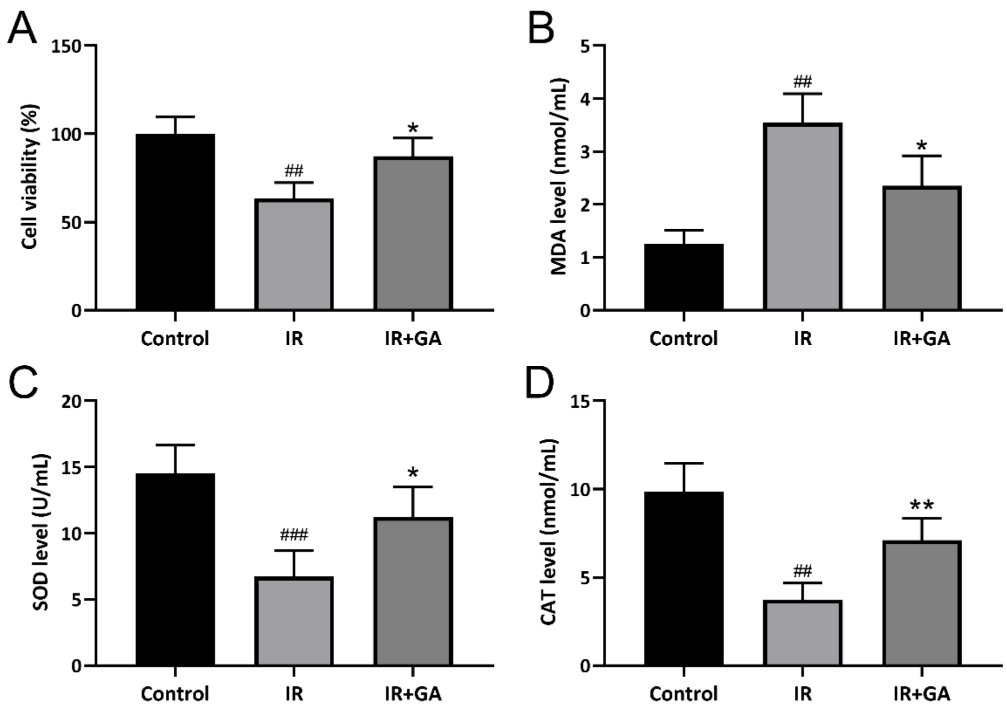

Figure S2. Gallic acid attenuated ischemia reperfusion (IR) injury induced oxidative stress in human renal proximal tubular epithelial cell line (HK-2). HK-2 cells were treated with 10 mM gallic acid supplemented in the culture medium and then incubated for 12 h under the hypoxic condition containing 1% O_2, 94% N_2, and 5% CO_2 in medium without serum, followed by culturing in normal medium and normoxic for 24 h. The cell viability was measured by CCK8 (A). The levels of MDA (B), SOD (C) and CAT (D) in supernate were compared. Data are presented as mean ± SD. ##p < 0.01, ###p < 0.001 compared to control. *p < 0.05, **p < 0.01 compared to IR.

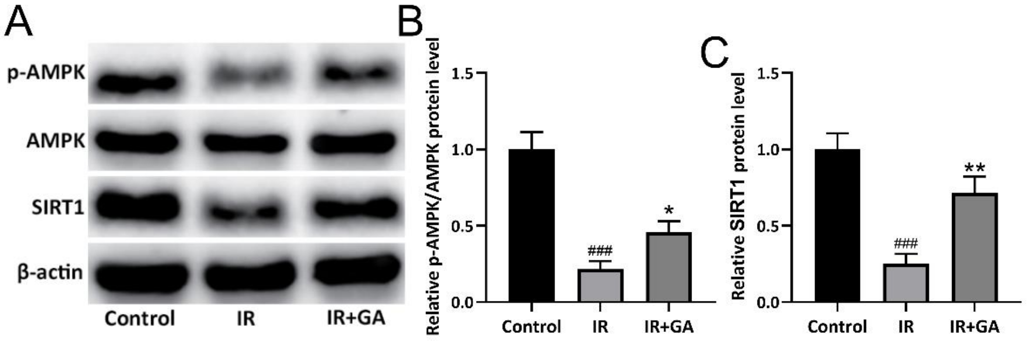

Figure S3. Gallic acid activated AMPK/SIRT1 signaling pathway in ischemia

reperfusion treated human renal proximal tubular epithelial cell line (HK-2). HK-2 cells were treated with 10 mM gallic acid supplemented in the culture medium and then incubated for 12 h under the hypoxic condition containing 1% O_2, 94% N_2, and 5% CO_2 in medium without serum, followed by culturing in normal medium and normoxic for 24 h. Western blotting was used to measure the protein expressions of p-AMPK, AMPK and SIRT1 (A) and the relative expressions were normalized to control (B and C). β-actin was used as a loading control. Data are presented as mean ± SD. ###$p < 0.001$ compared to control. *$p < 0.05$, **$p < 0.01$ compared to IR.

Information Classification: General
